# An approach to the management of acute poisoning in emergency settings

**DOI:** 10.4102/safp.v66i1.5841

**Published:** 2024-02-27

**Authors:** Ramprakash Kaswa

**Affiliations:** 1 Department of Family Medicine and Rural Health, Faculty of Health Sciences, Walter Sisulu University, Mthatha, South Africa; 2 Department of Health, Mthatha Regional Hospital, Mthatha, South Africa

**Keywords:** poison, toxic substances, absorption, antidote, elimination

## Abstract

The impact of poisoning can differ significantly depending on the specific substance consumed. Identifying toxic substances in a patient is crucial to obtaining a thorough medical history. Frontline healthcare providers in the emergency department often handle patients presenting with poisoning. Their clinical presentation can vary depending on their dose, duration of exposure, and pre-existing medical conditions. Initially, poisoning management entails administering supportive care such as absorption and enhancing the elimination of poison with charcoal and antidote administration after identifying the poisoning substances. This article aims to provide a basic overview of the concepts involved in evaluating and managing these individuals.

## Introduction

The word ‘poisoning’ denotes the deliberate or unintentional injury or impairment of a living organism by a substance through its chemical action. This concept is used to imply that accidental ‘Toxic exposure’ to a substance is unintentional, and ‘Toxic overdose’ refers to intentional or accidental exposure to a harmful substance.^1,2^ Even though the terms overdose and poisoning are often used interchangeably when discussing drugs of abuse, the former does not typically result in poisoning unless it triggers symptoms.^1^

In 2019, the Global Burden of Disease (GBD) study reported that unintentional poisoning accounted for 0.14% of all global deaths, while self-harm contributed 1.34%. Despite the low rate of death caused by this issue, the World Health Organization (WHO) has long stated that poisoning is a significant public health issue.^3^ Acute poisoning is a complex public health issue requiring prompt medical attention, resulting in the hospitalisation or death of thousands of individuals. According to the WHO, about 800 000 people die annually because of suicide and self-harm poisoning accounts for 1.34% of global deaths. The WHO considered poisoning a significant public health issue in middle- and low-income countries.^3,4^

The reported cases of poisoning and self-harm in South Africa mirror the international trends.^5^ The exact number of poisoning incidents is complex to ascertain because of the exclusion of fatalities resulting from drowning or trauma caused by intoxicated persons in reported data. Similarly, the long-term effects of chronic exposure to environmental toxins on human health are not reported.^6^ Providing adequate medical care to patients affected by poisoning is imperative, and healthcare providers should be encouraged to promptly report poisoning as a notifiable medical condition, such as lead, mercury, and agricultural or stock remedy poisoning.^7^ This article emphasises the importance of managing acute poisoning in emergency healthcare settings.

## Diagnosis of poisoning

### History

History-taking aims to identify the toxic substance that caused the overdose or poisoning. The comprehensive history of a poisoned individual is essential to understanding their symptoms, exposure, and circumstances. Healthcare providers should maintain a confident and calm demeanour to facilitate the exchange of information. If a patient cannot divulge a complete history, then an attempt for collateral information from their friends or family must be made.^1^

### Physical examination

Healthcare workers handling suspected individuals exposed to poisonous nerve gases or organophosphate pesticides must use appropriate protective equipment before performing any procedure. Healthcare workers are prone to being impacted by contaminants while working. A comprehensive physical examination is critical while dealing with patients suspected of being poisoned. It can help to identify the toxidromes and recommend the appropriate treatment.

## Major clinical signs and symptoms of toxicity (toxidromes)

### Level of consciousness

Reduced level of consciousness is one of the most common symptoms of acute poisoning. Before diagnosing poisoning, the physician must rule out other systemic conditions such as diabetes, syncope, stroke, head injury, epilepsy, meningitis, encephalopathy, among others.^1,8,9^

### Pupil reflexes

Poisoning substances can be diagnosed by evaluation of pupillary reflex reactions. For instance, dilated pupils (mydriasis) can indicate cocaine, amphetamines, cannabis, and antidepressants. On the other hand, pinpoint pupils (miosis) can suggest an overdose of various drugs, such as opiates, organophosphate, and chloral hydrate.^1,4^

### Eye movement

Acquired spontaneous eye movements (nystagmus) are commonly caused by acute drug poisoning. Various drugs, such as phencyclidine, ethanol, and phenytoin can cause nystagmus.^8,9^

### Breathing

It is essential to observe the unusual breathing patterns of patients. For instance, any disorder leading to significant acidosis can trigger Kussmaul respiration, commonly occurring with alcohol poisoning or salicylate toxicity. In addition, compensating hyperventilation can accompany metabolic acidosis or methanol poisoning. In some cases, a central nervous system depressant used by a patient can trigger a respiratory arrest.^1,4^

### Motor function

It is important to remember that patients who have been poisoned or overdosed on drugs or alcohol may have flaccid paralysis. They may also have dilated pupils, are unresponsive, and have no pupil deviation in response to cold-water injection into the ear canal (cold calorie test). They can recover fully even though they have been in a coma.^1^

### Cardiac arrhythmias

Continuous electrocardiography is essential for patients with significant poisoning. It can reveal critical diagnostic signs, such as arrhythmia in cocaine and theophylline toxicity, prolonged PR interval (beginning of the P wave until the beginning of the QRS complex) in digoxin overdose, a widened QRS complex (beginning of the Q wave to the end of the S wave) during an antidepressant overdose, or prolonged QT intervals (beginning of the Q wave to the end of the T wave) following an arsenic poisoning.^1,2^

### Seizures

Poisoning and drug overdose can induce a seizure. Delayed seizures may indicate alcohol withdrawal or sedative-hypnotic withdrawal. For people who have overdosed on an unknown substance, the standard seizure control method is to use the full dosage of benzodiazepines.^1^

### Gastrointestinal disturbance

Some of the common causes of gastrointestinal upset because of toxic ingestion include prolonged vomiting (theophylline) and gastrointestinal bleeding (iron and arsenic toxicity). Other conditions, such as arsenic and lithium poisoning, can lead to massive diarrhoea. In acute mercury poisoning, patients have a mucous-type diarrhoea that can lead to haemorrhagic colitis.

## Diagnostic tests

**FIGURE 1:**
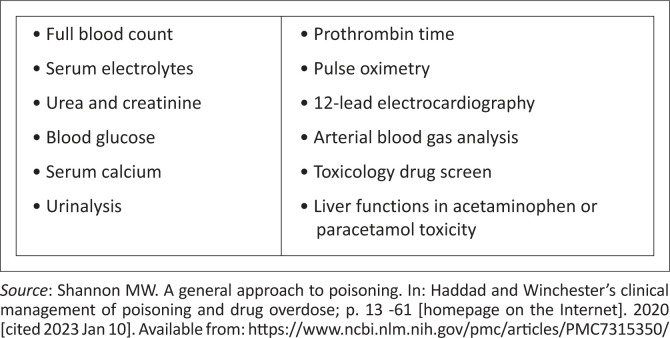
Common diagnostic test for acute poisoning case.

The treatment of poisoning victims determined by laboratory tests. The standard tests required for every significant poisoning are listed in Figure 1.^1^ Although it is essential to remember to treat the patient first, not the laboratory results. Therapy should not be withheld while waiting for the drug level to be confirmed in critical patients.^8^

## Vitals stabilisation

**FIGURE 2:**
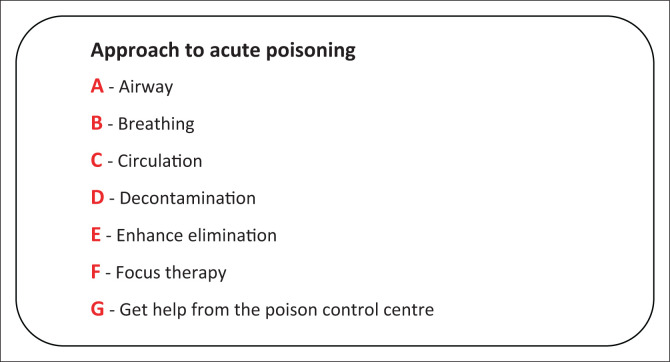
Approach to the poisoning case in emergency room.

A patient present with poisoning is usually unstable; one must, first of all, stabilise their vitals and have an airway, adequate ventilation, and the maintenance of circulation described in Figure 2.^10,11^ Exposure to certain types of chemicals or drugs can cause respiratory tract compromise and needs rapid sequence intubation and ventilator support. Some of these include corrosive ingestion and chlorine inhalation. Other conditions include pulmonary oedema (inhalation injury), bronchorrhea (organophosphates), central nervous system (CNS) depression (opioids, barbiturates, alcohols), seizure (theophylline, isoniazid), and aspiration described in Figure 2.^1,2^ Patients declared poisoned usually arrive at the emergency room with cardiogenic shock or hypotension. The low blood pressure caused by an ingested substance can vary depending on its nature.^2^ For instance, it can be caused by a variety of factors, such as the effects of a drug on the heart’s contractility (β-blockers, calcium-channel blockers, clonidine); conditions such as gastrointestinal fluid losses (heavy metals, mushrooms) and peripheral vasodilation (angiotensin-converting enzyme inhibitors) can also cause hypotension.^1,8^ The tissue perfusion and blood pressure should be maintained by administering vasopressor drugs (norepinephrine, epinephrine, dobutamine, phenylephrine, and glucagon) and volume augmentation.^8^

## Decontamination

### Skin and eyes

Water is frequently utilised as a skin and eye decontaminant agent, effectively minimising the effects of dermal exposure.^1^ In the case of skin burns caused by toxin exposure, it is necessary to follow established burn management procedures. These include the use of wound dressings and monitoring for infection.

### Gastrointestinal tract

#### Pre-absorption elimination

**FIGURE 3:**
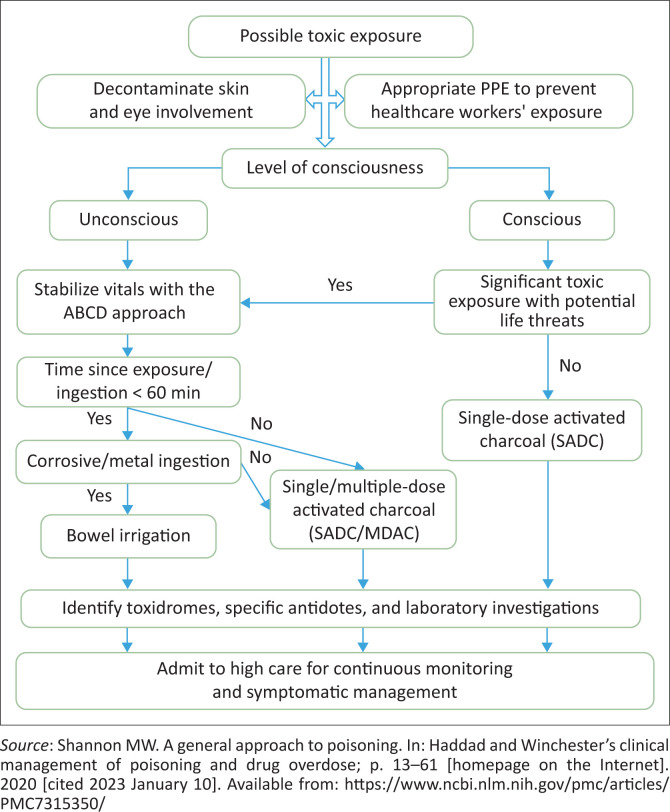
The flow diagram for acute poisoning management.

The decisions related to the decontamination of poisoning should be individualised. Figure 3 shows a suggested algorithm and should be considered a starting point for considering various options.^1^

### Activated charcoal

Activated charcoal binds different substances, making them less accessible for systemic absorption in the gastrointestinal tract. It is effective if administered within an hour after ingestion.^8^ The recommended dose of activated charcoal is 0.5 g – 1 g in children and 25 g – 100 g in adults. Multiple-dose activated charcoal is often used to eliminate life-threatening toxic doses of substances that undergo enteroenteric or enterohepatic circulation.^1^ Sorbitol-containing charcoal should be avoided as it is emetic and causes dehydration in infants. Those with compromised airways or a reduced consciousness level are not a candidate to use charcoal until intubation. It is not advisable in some instances, caused by ingesting acids or alkalis, heavy metals, or alcohol.^8^

### Whole bowel irrigation

A large amount of polyethylene glycol is administered through either mouth or nasogastric tube. The goal of this procedure is to remove the toxic substance from the rectal tract in a fast and efficient manner. One of the main reasons this method is commonly used is when poisonous substances such as lithium and iron or enteric-coated drugs that are poorly absorbed by activated charcoal, and in case of heroin, cocaine, and opioids packet ingestion (body packing or stuffing).^1,4^ The nasogastric or mouth tube delivers the polyethylene glycol solution at 25 mL/kg/hr – 40 mL/kg/hr until the effluent from the rectal tract is clear. Individuals with gastrointestinal bleeding or mechanical or functional bowel obstructions are excluded from whole bowel irrigation. It should also be avoided in unstable conditions where the airway cannot be protected.^1^

### Gastric lavage

Gastric lavage is highly controversial and can vary depending on the practitioner’s training and background. A gastric lavage is a procedure that involves blind placement of a large-bore tube into the stomach. It can cause various complications, including hypoxia and perforation of the gastrointestinal tract. In addition, it can lead to aspiration pneumonitis. The main indication for the procedure is to prevent severe harm caused by the substances ingested within an hour. Ingestion of petroleum products, acidic substances, and the inability to protect one’s airway are contraindications to the gastric lavage procedure.^1,11^

### Post-absorption elimination

Several theories are presented to explain the accelerated clearance of toxins after they have been absorbed into the gastrointestinal tract. Firstly, using multidose-activated charcoal disrupts the enterohepatic circulation. The procedure is commonly known as ‘gastrointestinal dialysis’.^1^ Secondly, only utilise the extracorporeal methods (peritoneal dialysis, haemodialysis, and haemoperfusion) for poisonings predicted to result in toxic metabolic activation and severe metabolic acidosis, especially when treatment modalities are ineffective. This includes those involving ethylene glycol and methanol and those not responding to treatment despite being given the necessary support.^9^

## Antidote administration

**FIGURE 4:**
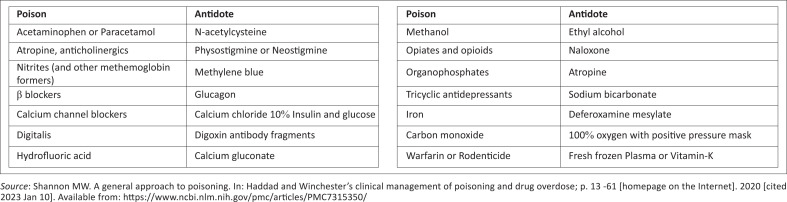
Common poisoning substances and their antidotes in clinical toxicology.

The availability of sophisticated antidotes and their use have become essential in clinical toxicology. In most cases, an initial use of an antidote is recommended for stabilisation within the first hour following exposure to a known substance. Figure 4 summarises the most common poisons and their antidotes.^8^

## Monitoring and disposition

Individuals who have experienced poisoning require symptomatic care and monitoring. The specific toxic substance is often unknown, and for the patient with severe poisoning, it is crucial to closely monitor the patient’s oxygen saturation, vital signs, and cardiac rhythm.^2^ Unstable patients whose airways cannot be protected may need to be transferred to a high-care or intensive care unit for continuous monitoring and ventilator support. A comprehensive monitoring strategy, which includes a multidisciplinary team, is highly recommended.^5^

It is imperative for individuals who have underlying health conditions, such as diabetes or heart failure, to receive close monitoring following a poisoning. This is crucial as the poisoning may have exacerbated their conditions.^9^ Moreover, observation may be indispensable in addressing their injuries from the overdose. The decision of disposition of a patient who has intentionally self-poisoned must be made according to their psychosocial disorder status. Before their release, all patients with this type of disorder must undergo a psychosocial assessment.^2,5^

## Conclusion

In primary care, cases of poisoning require close observation, whereas severe toxicity demands urgent intervention to establish the patient’s airway, breathing, and circulation. Healthcare providers gather information by analysing the patient’s history, conducting laboratory tests, and assessing for toxidromes to identify the poisoning substance. Once the specific poison substance is determined, appropriate antidotes should be administered for treatment.
